# Indoor Visual-Based Localization System for Multi-Rotor UAVs

**DOI:** 10.3390/s22155798

**Published:** 2022-08-03

**Authors:** Massimiliano Bertoni, Stefano Michieletto, Roberto Oboe, Giulia Michieletto

**Affiliations:** Department of Management and Engineering, University of Padova, Stradella S. Nicola, 3, 36100 Vicenza, Italy; stefano.michieletto@unipd.it (S.M.); roberto.oboe@unipd.it (R.O.); giulia.michieletto@unipd.it (G.M.)

**Keywords:** indoor localization, aerial vehicles, Visual Inertial Odometry, fiducial markers

## Abstract

Industry 4.0, smart homes, and the Internet of Things are boosting the employment of autonomous aerial vehicles in indoor environments, where localization is still challenging, especially in the case of close and cluttered areas. In this paper, we propose a Visual Inertial Odometry localization method based on fiducial markers. Our approach enables multi-rotor aerial vehicle navigation in indoor environments and tackles the most challenging aspects of image-based indoor localization. In particular, we focus on a proper and continuous pose estimation, working from take-off to landing, at several different flying altitudes. With this aim, we designed a map of fiducial markers that produces results that are both dense and heterogeneous. Narrowly placed tags lead to minimal information loss during rapid aerial movements while four different classes of marker size provide consistency when the camera zooms in or out according to the vehicle distance from the ground. We have validated our approach by comparing the output of the localization algorithm with the ground-truth information collected through an optoelectronic motion capture system, using two different platforms in different flying conditions. The results show that error mean and standard deviation can remain constantly lower than 0.11 m, so not degrading when the aerial vehicle increases its altitude and, therefore, strongly improving similar state-of-the-art solutions.

## 1. Introduction

A multi-rotor platform is a rotary wing Unmanned Aerial Vehicle (UAV), generally consisting of a rigid body, actuated by a set of spinning propellers, whose configuration determines the vehicle actuation properties [1]. In recent decades marked by the advent of Industry 4.0, smart homes, and  Internet of Things (IoT), multi-rotor UAVs have emerged as a leading and promising technology in civil, rural, and industrial contexts [2,3,4,5], where they are used in several applications, ranging from traditional surveillance and monitoring tasks [6,7] to modern contact-aware inspection and manipulation [8,9].

In most of the application scenarios, the multi-rotor platforms are required to localize themselves in the environment, namely to estimate their position with respect to an a priori fixed reference frame. Due to its high pervasiveness, such an issue constitutes a well-studied problem within the aerial robotic community and several solutions have been proposed in the literature. Most of the existing works focus on the outdoor localization of UAV, where the principal challenge rests on the global navigation satellite system (GNSS) signal (temporary) loss and/or degradation (see, e.g., [10,11,12]). Localization in indoor environments is, instead, less investigated, as it turns out to be a difficult issue per se, which contributes to hindering the spread of UAVs indoor applications. The indoor environment represents one of the main cases of GNSS-denied areas, mainly because the satellite signals are blocked or corrupted by physical barriers. As a consequence, multi-rotor UAVs are required to rely on a different data source to compute a position estimation, and thus they need to be equipped with other alternative (and possibly lightweight) sensors. In addition, a higher level of accuracy is generally required for position estimation when flying in physically limited and possibly cluttered areas. Despite these challenging aspects, recent IoT-inspired technological trends boost the employment of autonomous aerial vehicles to perform different tasks in indoor scenarios, especially in the emerging smart domestic and industrial contexts. These include, for instance, cooperative monitoring [13], ground robots tracking [14], infrastructures contact inspection [15], people and objects detection [16], items transportation [17].

In light of these facts, this work focuses on the indoor localization problem. We outline a UAV positioning strategy based on the design of a customized map of visual fiducial markers (namely AprilTag), which turns out to be capable of ensuring the position retrieval for different multi-rotor UAVs while flying at various altitudes.

### 1.1. Related Works

Since communication with the satellite network is partially or completely impractical in any indoor environment, for the UAVs acting in these contexts, other sources of position information are necessary, such as acoustic [18], radio frequency [19] signals, visible light communication [20], vision systems [21].

Vision systems, and in particular cameras, represent the most widely used technology when dealing with indoor localization [22,23], due to their robustness and the level of accuracy they can ensure. Along this line, several academic realities employ motion capture systems or other high-performance camera networks to track UAV movements [24]. These localization methods are very precise and typically can provide position data in real time; however, they represent very expensive and invasive solutions, not suitable, for instance, for industrial scenarios. For these reasons, hereafter, attention is focused on localization solutions, which rely on on-board vision systems, namely lightweight cameras mounted on the UAVs.

In this direction, most existing image-based positioning strategies are based on the detection and identification of some fiducial markers [25]. Taking into account the existing literature, this approach is principally exploited when coping with the (outdoor) precise autonomous landing: this maneuver, in fact, requires fairly accurate position data and thus cannot be fulfilled by resting only on GNSS signals when available. In this application scenario, the employed fiducial markers can be custom as, for example, in [26,27,28]. Alternatively, they can belong to some standardized families, namely sets of *fiducials* consisting of patterns artificially designed to simplify detection by using a camera. Adopting this approach, a visual-servo UAV landing control strategy is proposed and validated through simulation results in [29] and then tested on a real aerial platform in [30]. Beyond the precise landing, several state-of-the-art solutions take advantage of the vision-based approach to deal with target identification. For example, in [31,32] UAV landing is preceded by the localization of a target associated with a certain fiducial tag. Similarly, in [33,34], the UAV detection of (and eventually the reaction to) multiple targets is taken into account, based on the exploitation of different fiducial markers. Furthermore, in [35], the tracking of a ground robot by a UAV is performed through tag detection. Likewise, the tracking of another flying UAV is considered in [36].

Visual information is frequently combined with the data gathered by other onboard sensors. In [37,38], for instance, both Inertial Measurement Unit (IMU) and GNSS signals are also taken into account to cope with UAV landing on a moving surface, and a similar sensor fusion approach is described in [39] for landing on a static pad. Both cameras and IMUs are often already mounted on all kinds of mobile robots since they are cheap all-purpose sensors. In such context, visual input and inertial information are used to estimate pose and velocity in real time. The process of localizing something by considering the camera and IMU sensors only is usually called Visual Inertial Odometry (VIO) [40]. VIO enables accurate localization without using systems based on GNSS or Laser Imaging, Detection, and Ranging (LIDAR) solutions. Thus, it turns out to be a viable strategy in scenarios where satellite signals are unreliable or not available and/or LIDAR is not accessible due to costs or payload limits. In light of these facts, VIO constitutes the ideal candidate for indoor localization for the majority of aerial vehicles.

A notable issue, generally affecting the fiducial marker-based solutions, concerns the need to properly see such markers. This implies some constraints on UAV altitude which determine the distance between the camera and the markers but also affects the field of view of the camera, thus conditioning the overall quality of the detection. This drawback is faced in [41] by combining the IMU data and the information retrieved by a simplified marker when the vehicle altitude drops below a certain threshold. Other possible strategies consist of including multiple smaller and complete markers within a larger one [42,43], or using distinct fiducial markers of different sizes [44,45].

On the other hand, the literature related to UAVs navigation based on the detection and identification of tags belonging to standardized families is very poor. This is probably due to the need to find the suitable selection and placement of fiducial markers depending both on the considered aerial platform and the required task (determining the flying conditions in terms of maneuver dynamism and altitude level). In this direction, a ground map generated from a simple and repetitive pattern composed of equally sized tags is proposed in [46] to navigate a Bebop2 quadrotor equipped with a monocular camera. The experimental results show that by processing the position data collected by the vision-based system through a Kalman Filter (KF), the error in the pose estimation is very small (±0.02 m in the *x* and *y* position component when flying in a 2 × 2 m${}^{2}$ area) as compared with the output of a motion capture system; nevertheless, the investigated flying maneuvers are characterized by constant altitude. A similar framework is discussed in [47], replacing the ground map with a scattered set of markers placed at some points in the environment whose location is known. In this case, focusing on planar movements, the experimental tests reveal that the position estimation error increases concurrently with the distance of the UAV from a marker. To mitigate this fact, in [48] the acquired position data is fused with the IMU measurements using an Extended Kalman Filter (EKF) approach: on the (x,y) plane, the error range reduces from [−1.0, 0.6] m to [−0.2, 0.6] m, and localization accuracy increases as the UAV gets closer to the tags.

### 1.2. Contributions

From the existing literature, it emerges that the most challenging aspects of image-based indoor localization are related to the need to ensure the proper and continuous detection and identification of (at least) a fiducial marker, (possibly) independently of its distance. In the case of UAVs navigation, such a requirement results in being particularly demanding due to the intrinsic unstable nature of the dynamics of aerial platforms, also characterized by a high sensibility to external noise and unexpected events.

Taking into account these facts, in this work we describe a VIO localization method based on fiducial markers, which aims at ensuring the navigation of any multi-rotor UAV vehicle in an indoor environment. In particular, motivated by the increasing number of industrial applications involving aerial platforms, we account for a quasi-static context whose topology allows the presence of a wide planar map placed either on the ground or on the ceiling. One of the original aspects of the proposed navigation solution, indeed, consists in the design of a map of fiducial markers having the following features:

- *Density*—the map counts a high number of narrowly placed tags;
- *Size heterogeneity*—the map is made up of fiducials belonging to four different classes in terms of size.

The first feature is justified by the intent of limiting as much as possible the loss of updated position information during UAV flight to cope with the complex dynamics of the aerial platforms and to ensure a certain level of safety. The employment of markers with different sizes is, instead, motivated by the purpose of proposing a localization method valid for (different) multi-rotor platforms flying at various altitudes, guaranteeing high estimation quality. In this sense, the designed positioning method also allows us to cope with take-off and landing maneuvers during which the UAV altitude changes quite rapidly. In this sense, as compared to most of the state-of-the-art works, we provide a localization system that turns out to be suitable for the whole UAV navigation task, ensuring the same level of positioning accuracy for the take off, landing and more complex maneuvers.

Inspired by [48] and similar solutions, we adopt an EKF approach to fusing the position data derived by the marker-based localization method and the inertial measurements acquired by some onboard IMU sensors. We access the performance of such an indoor localization solution through an experimental testing campaign wherein the output of a VICON motion capture system is assumed as ground truth. In particular, overcoming the results of [48], we focus on both planar and vertical movements, thus discussing the performance of the outlined strategy as a function of the distance from the tags but also in the case of rapid longitudinal maneuvers between two different points. Moreover, we test the outlined strategy on both a small-medium size custom quadrotor and a medium-large size custom hexarotor, sharing the same flight controller and sensing system. This represents another original aspect with respect to the existing literature, wherein typically the tests are conducted on a single aerial platform. Finally, we highlight that the entire experimental setup is based on a cutting-edge ROS2 architecture.

### 1.3. Paper Structure

The rest of the paper is organized as follows. Section 2 is devoted to the problem statement: we provide an overview on the considered UAV vehicles and on the EKF-based sensor fusion. Section 3 presents the outlined VIO localization method, describing its ROS2 implementation and focusing on the design of the fiducial map. The performance of the outlined localization solution is discussed in Section 4 which is devoted to the conducted experimental campaign. The main conclusions are drawn in Section 5.

## 2. Problem Statement

In this work, mainly motivated by the emerging trend of integrating the UAV presence within industrial productive processes, we aim at proposing an efficient solution for the localization of the popular star-shaped multi-rotor platforms. In the rest of this section, we provide an insight into the modeling and control of such aerial vehicles (Section 2.1) and then we briefly describe the rationale that guides the sensor fusion process based on the EKF approach (Section 2.2).

### 2.1. Star-Shaped Multi-Rotor Modeling and Control

Due to their high maneuverability and simple structure, quadrotors represent the most popular and currently used aerial platforms. Nevertheless, very recently, the robotic community has focused its attention towards the design and exploitation of new aerial platforms having more than four rotors, with the purpose of improving the actuation properties (we refer the reader to [1] for a proper and complete taxonomy of multi-rotor platforms). Both standard quadrotors and most of the studied hexarotor and octorotor platforms are characterized by a *star-shaped* configuration, where the rotors are evenly spaced on a circumference centered in the vehicle center of mass (CoM) and spin about parallel axes in opposite direction with respect to their neighbors [49].

Independently of their number, while spinning the propellers of any star-shaped multi-rotor generate some *thrust forces* and a *drag torques*. The combination of those quantities then results in the total *control force* and *control torque* exerted on the vehicle CoM. More formally, taking into account a global fixed reference frame (*world frame* $F_{W}$) and a local frame centered in the vehicle CoM (*body frame*$F_{B}$), the dynamics of any star-shaped multi-rotor can be described through the Newton-Euler approach. Thus, we have the following model:

(1a) $$\begin{array}{cc} m{\ddot{p}}_{W} & =mgz_{W}{+}^{W}R_{B}f_{B}^{c}z_{B}, \end{array}$$

(1b) $$\begin{array}{cc} m{\dot{\omega }}_{B} & =-\omega_{B}\times J\omega_{B}+\tau_{B}^{c}, \end{array}$$

where $m,g\in R^{+}$ denote the vehicle mass and the gravity constant, respectively, the (positive definite) matrix $J\in R^{3\times 3}$ represents the inertia of the vehicle calculated in $F_{B}$, and the versors $z_{W},z_{B}\in R^{3}$ identify the direction of the *z*-axis of the world frame and of the body frame, respectively. The vector $\tau_{B}^{c}\in R^{3}$ in (1) denotes the control torque expressed in $F_{B}$, while the scalar $f_{B}^{c}\in R$ represents the intensity of the control force, also expressed in $F_{B}$. Finally, $p_{W}\in R^{3}$ identifies the position of the vehicle in $F_{W}$ and $\omega_{B}\in R^{3}$ is its angular velocity expressed in the body frame, and the rotation matrix ${}^{W}R_{B}\in SO\left(3\right)$ describes the orientation of $F_{B}$ with respect to $F_{W}$, namely the attitude of the UAV in the world frame. Hereafter, we refer to the pair ($p_{W}{,}^{W}R_{B}$) as the UAV state.

**Figure 1:**
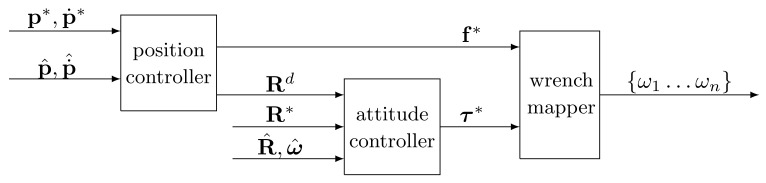
Typical cascaded controller structure: subscripts indicating the reference frames have been removed for sake of clarity.

We remark that the star-shaped multi-rotor platforms are characterized by four controllable degrees of freedom (consisting in $f_{B}^{c}$ and $\tau_{B}^{c}$), thus they are under-actuated systems. Furthermore, the presence of ${}^{W}R_{B}$ in (1a) also implies the existence of a strong coupling between the translational and rotational dynamics of the platform. For these reasons, the dynamics of any star-shaped multi-rotor can be regulated by exploiting the well-known *cascaded control approach* (see, e.g., [50,51]). Such a motion regulation strategy entails the presence of two control loops: an outer and slower position control loop and an inner and faster attitude control loop (Figure 1). Both the position and attitude controllers generally consist of one or more suitably tuned PID regulators whose gains need to be selected by considering the specific multi-rotor platforms. However, we draw attention to the fact that the effectiveness of a cascaded control scheme is highly dependent on the accuracy of the feedback information; in particular, it is necessary to retrieve a good estimation of the current position and attitude of the vehicle.

### 2.2. Kalman-Based Sensor Fusion

Aerial platforms account for some limitations in terms of transportable payloads and also computational capabilities. Hence, the estimation of the UAV position and attitude is often performed by exploiting the redundancy of the noisy information collected by different lightweight sensors. Along this line, several well-stated techniques for indoor and outdoor UAV localization take advantage of using IMU measurements. Typically, IMU sensors consist of a 3-axes gyroscope and a 3-axes accelerometer, and often also a 3-axes magnetometer is included. We underline that IMU devices turn out to be particularly advantageous in aerial robotic navigation due to their compact design and robustness against malicious attacks due to the fact that the gathered measurements are retrieved in a local (body) frame without relying on external sources (as in the GNSS case). However, the recorded angular velocity and linear acceleration data are usually noisy and affected by constant bias; thus, the state estimation that can be derived by exploiting the IMU data is usually affected by a high level of inaccuracy and is also characterized by drifts.

To deal with these drawbacks, IMU measurements are often combined with other position data, gathered by various sensing devices, and in most cases, the information fusion is performed using a Kalman approach. This filtering technique, in fact, allows one to properly integrate all available position and attitude measurements by accounting for vehicle dynamics (1). In this work, we account for an EKF able to provide a suitable estimate of the state (pose) of the vehicle and also its linear velocity, jointly also with the IMU measurement bias and the wind velocity. We emphasize that the adopted EKF is capable of combining sensor data with different delays and rates in a mathematically consistent way. In detail, hereafter, we assume that the considered star-shaped multi-rotor is equipped with a camera that provides information at a certain rate, lower with respect to the IMU data acquisition frequency. The data gathered by both the IMU and the camera constitute the inputs of the EKF.

## 3. VIO Localization

**Figure 2:**
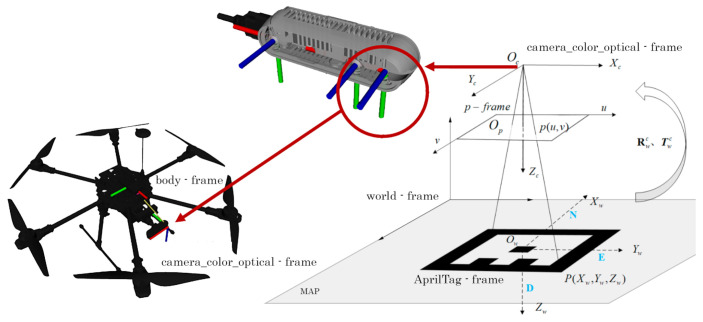
Tag-based localization: this localization method is based on the identification of the roto-translation between the reference frame associated with the detected fiducial marker (*AprilTag-frame*) and the reference frame in-built with the camera mounted on the aerial platform (*camera_color_optimal-frame*) having known roto-translation with respect to the UAV body frame.

We propose a VIO localization solution based on an EKF merging information from visual fiducial marker detection and relative motion obtained through two IMUs redundant systems. The UAV state is estimated at a high frame rate (∼60 fps) by using the last information from the considered sensors. We highlight the fact that visual fiducial markers, also called fiducials, are artificial patterns designed to simplify the detection of data bits by using peculiar shapes, usually black or white squares. Fiducial families consist of several tags that correspond to different patterns. Each tag represents a coordinate frame in the world that is used to easily identify the pose of the camera on board the vehicle, and therefore its state. The algorithm relies on a set of geometric constraints such as tag shape, dimension, and flatness to compute the 3D pose of all visible fiducials with respect to the camera based on the information within the image plane (Figure 2).

The localization system is designed to (i) collect images from the camera, (ii) detect all the available fiducials in the scene, (iii) interpret the data bits in each marker, and (iv) compute the UAV state relating the data to the map information. Among the wide variety of fiducial markers [25], we select AprilTag [52] for three main reasons: the good performance at a higher distance (or smaller tags), the larger bit pitch at the same physical size, meaning families with many more possible tags, and finally the robustness to detection errors due to a wide hamming distance between tags.

### 3.1. Map Definition

We develop a method to create fiducial maps composed of a large set of tags. The tag positioning is defined to maximize the number of visible markers in the scene under various camera characteristics and flying conditions. Consequently, this corresponds to maximizing localization capabilities on different aerial platforms. The actual localization space depends on the UAV features. In particular, the flying height is strongly affected by camera characteristics since they influence the tag detecting range.

In this work, we account for the camera impact minimization by selecting a set of four physical dimensions for tags, namely S ($5.75\;cm^{2}$), M ($11.5\;cm^{2}$), L ($23\;cm^{2}$), XL ($46\;cm^{2}$). We remark that there does not exist a standardization for tag dimensions which instead depends on the application context; hence, we select these sizes by an iterative experimental process to guarantee clear tag visibility of at least one tag size at different camera heights. In detail, the three main camera factors that guide size selection are field of view, resolution, and focus.

**Figure 3:**
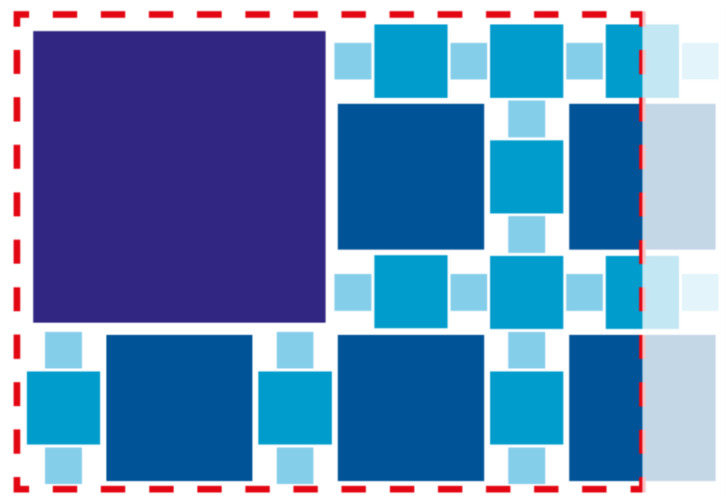
Basic tag design: this pattern has been used to generate the fiducial map by applying roto-translation and mirroring operations.

Then, given that we account for a dense map, the fiducial family derives directly from the number of tags necessary to fill the vehicle working area. More in detail, we identify the pattern in Figure 3 where markers of different sizes are suitably alternated in order to cover all the available space guaranteeing good tag detection at different heights. This basic design repeats after roto-translation and mirroring operations to compose the generated map. Note that, in this way, a nominal map area of 5 m×7 m requires 772 S tags, 526 M tags, 212 L tags and 35 XL tags, for a total amount of 1545 tags. This implies the use of the fiducial family *TagStandard41h12* composed of 2115 tags.

Any tag in the map is associated with a reference frame (*AprilTag frame*) placed in the tag center and has the *z*-axis pointing downward (as depicted in Figure 2) likewise the body frame $F_{B}$ in-build with the UAV. All the tag frames result to be aligned among them. We also assume that the world frame $F_{W}$ is located in a corner of the map and it is oriented so that it’s *z*-axis points upward, while the *y*-axis is concordant with those of the tags reference frame. Tag-based localization entails the identification of the roto-translation between the reference frame associated with the bigger detected tag and the reference frame in-built with the camera mounted on the aerial platform whose pose (position and orientation) in $F_{B}$ is known from the UAV structural model.

### 3.2. Ros2 Implementation

Practical implementation of the outlined positioning method exploits the ROS2 middleware. The core VIO localization is made up of three nodes: (i) a camera driver, (ii) a fiducial marker detector, and (iii) a visual odometry estimator. The specific implementation of each node depends on the actual hardware mounted on the UAV platform, but all nodes share standardized input/output messages plus their peculiar interface.

**Figure 4:**
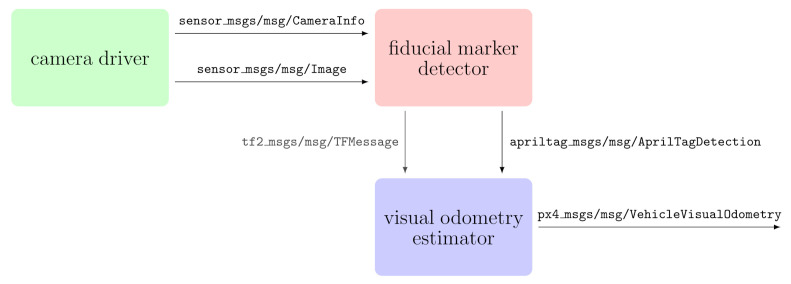
ROS2 architecture: ROS2 implementation of the proposed VIO localization strategy involves three principal nodes: camera driver, fiducial marker detector, visual odometry estimator.

The camera driver is the node controlling the camera settings and providing the camera information and the images collected by the sensor. Each node implementation is required to publish a sensor_msgs/msg/CameraInfo message to convey camera information and a sensor_msgs/msg/Image message containing image data. The fiducial marker detector subscribes to the camera driver output to identify the pose of the tags in the scene. The detection process looks for each tag according to fiducial family, id, and size. The main output is an array of apriltag_msgs/msg/AprilTagDetection messages. Optionally, the node can broadcast a transformation frame (tf2_msgs/msg/TFMessage) for each detected tag as part of the tf topic. The visual odometry estimator computes the vehicle position and attitude starting from the detected tags. Localization can consider a standalone marker or a tag bundle depending on the node policy and the detection accuracy. The output is a px4_msgs/msg/VehicleVisualOdometry message delivering the visual component of the VIO localization system. EKF will merge this result with the data recorded by IMUs. Figure 4 reports a schematic representation of the described architecture.

## 4. Validation

To assess the performances of the VIO localization method introduced in Section 3, we conduct an experimental campaign involving two different aerial platforms which fly in a 5 m×7 m×3 m indoor flight arena whose floor is covered with a 3.5 m×3.5 m AprilTag map structured as described in Section 3.1. In detail, the UAV operative space is restricted to the map area, where no obstacle is present.

### 4.1. Experimental Setup

**Figure 5:**
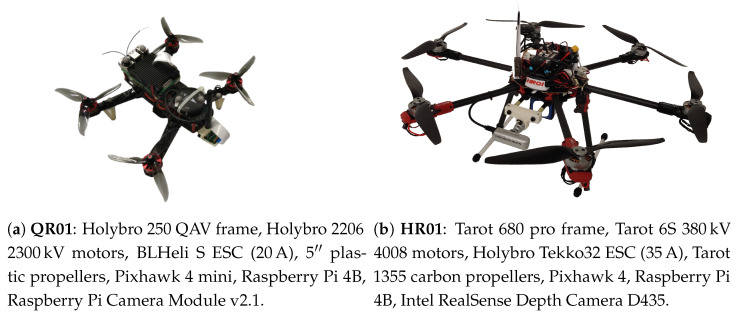
Star-shaped multi-rotor UAVs used for validation: (**a**) a small-size quadrotor having mass approximately 0.5 kg and (**b**) a medium size hexarotor having mass approximately 3.5 kg.

As far as the aerial platforms are concerned, we account for two star-shaped multi-rotor UAVs, a small-size quadrotor (QR01, in Figure 5a, having a diameter of ∼0.3 m) and a medium-size hexarotor (HR01 in Figure 5b, having a diameter of ∼0.8 m), both of them designed at the C-square lab of the University of Padova. In addition, the experimental setup involves a ground working station used for data logging and visualization, and a VICON motion capture system made of 10 cameras which provides the ground truth data for the UAVs localization working at 100Hz and having submillimeter accuracy.

Both HR01 and QR01 are equipped with a Pixhawk 4 flight controller and a Raspberry Pi 4B held in a light aluminum case with embedded fans. The former includes two redundant STMicroelectronics Flight Management Units (FMUs) based on an ARM Cortex M7 32 bit with the NuttX real-time operating system (RTOS), SI2MB Flash Memory and 512 kB RAM. The latter, instead, involves a quad-core ARM Cortex-A72 having 8 GB RAM. The choice of using a Raspberry Pi 4B as on-board companion computer is motivated by its small size (56×85×15mm), low-power consumption (3.8–4.0 W), and affordable price (≤$100) [53].

**Figure 6:**
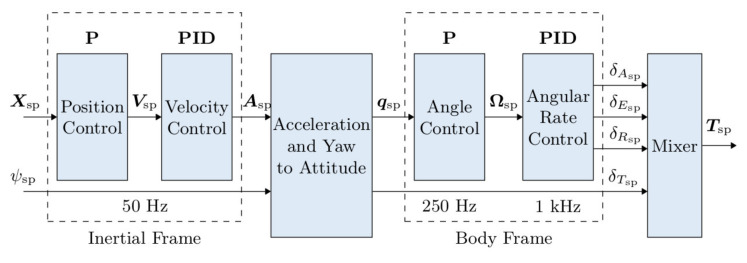
PX4 Autopilot internal control architecture: the inputs of the whole control block are the estimated position and yaw angle of the vehicle, the output is the set of duty cycles to impose to the actuators for realizing the computed normalized force commands, expressed as aileron, elevator, rudder and thrust. Note that the attitude is modeled adopting the quaternion convention.

**Figure 7:**
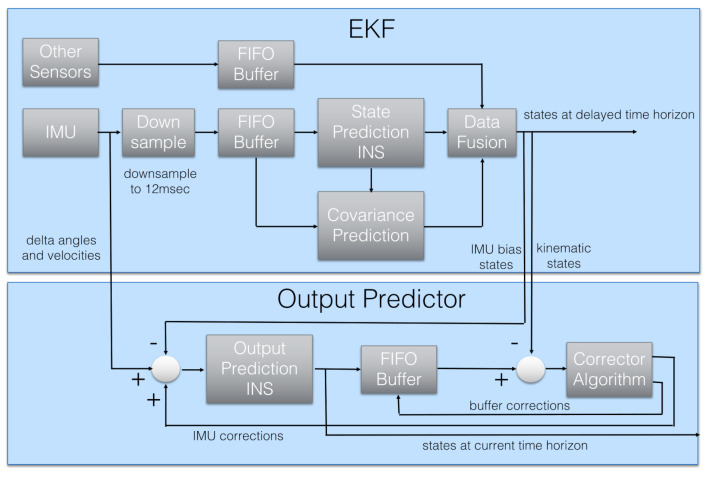
PX4 Autopilot EKF and Output Predictor internal architecture: Output Predictor guarantees the state prediction without delays and the concurrent EKF estimation is exploited to correct the resulting prediction. When accounting for the measurements of both the IMU sensors in the flight controller, two EKF instances run in parallel, and a selector compares the internal coherence of each one and determines the best sensors mix in terms of data consistency.

The Raspberry is responsible for handling the tag detection and identification process, the resulting output is then passed to the flight controller through serial communication. Based on PX4 Autopilot software, the flight controller elaborates the motor commands in light of the UAV state estimated via VIO localization approach (Figure 6). In particular, the position and attitude data extracted by fiducials are combined with the measurements recorded by the Pixhawk sensor set, which includes a couple of 6-axes IMU sensor and a barometric sensor, by means of a suitable EKF (Figure 7) whose working principles are detailed in [54].

Since we account for an image-based positioning strategy, the considered multi-rotor UAVs is also equipped with a camera, placed in front of the platform and pointing to the ground. In detail, QR01 mounts a 8-megapixel Raspberry Pi Camera module v2.1, whose resolution is up to 1920 p×1080 p ensuring a maximum frame rate of 30fps and Field of View (FOV) of $62^{\circ }\times 49^{\circ }$. An Intel Realsense Depth Camera D435 is, instead, on-board on HR01: this is characterized by RGB images acquisition with maximum resolution of 1920 p×1080 p, frame rate of 30fps and Field of View (FOV) of $69^{\circ }\times 42^{\circ }$. In both cases, we use a camera resolution of 320 p×240 p at maximum framerate during the experimental campaign. These framerate choices guarantee that frequency of the output of the VIO localization procedure is approximately 4Hz, in accordance with the typical rate of GNSS system.

### 4.2. Experiments Design

We conduct two sets of experiments on both the UAVs described in Section 4.1. These two sets distinguish the trajectories that the vehicles are required to follow resting on the PX4 off-board control flight mode. The selection of this autonomous flight mode is motivated by the possibility of realizing multiple trials in a replicable way, since it envisages the imposition of some way-points in terms of both position and yaw angle. In detail, each set of experiments accounts for five trials in order to have statistical consistency in the collected data.

The two considered trajectories consist of a planar square path along which the vehicle is required to keep a constant height from the ground (T1), and a sequence of vertical steps while maintaining the same position on the horizontal plane (T2).

#### 4.2.1. T1: Planar Square Trajectory

When T1 is taken into account, the UAV has first to execute the take-off maneuver in order to reach the starting position located at the height of 0.8 m from the ground. Then, it is successively required to

- Move forward (F phase—movement along the negative direction of *y*-axis of world frame);
- Translate along the orthogonal direction on the right (R phase—movement along the negative direction of *x*-axis of world frame);
- Move backward (B phase—movement along the positive direction of *y*-axis of world frame);
- and finally, translate along the orthogonal direction on the left (L phase—movement along the positive direction of *x*-axis of world frame) returning to the starting point.

Since T1 consists of a planar square trajectory, the traveled distance during each phase is the same and it is equal to 1.8 m. A static hovering phase is imposed at each corner of the square: the vehicle is required to maintain a fixed position and orientation for 10 s. The yaw reference, instead, is kept constant to zero during the whole path.

We consider the described trajectory because of two reasons. We aim at investigating the position information loss during rapid longitudinal and latitudinal movements (and the introduction of the hovering phases allows us to better distinguish each motion phase during the analysis). On the other side, T1 represents a path that can be executed by an aerial platform employed in a surveillance task of an indoor production area: the UAV can be in charge of monitoring different spatially distributed production stages while flying at a constant height because of safety constraints.

#### 4.2.2. T2: Vertical Steps Trajectory

In correspondence to T2, the UAV position on the (x,y)-plane of the world frame is required to remain the same during all the experiments. On the other side, after the take-off maneuver and a hovering phase of 10 s, a sequence of reference steps is imposed along the *z*-axis of the world frame: the vehicle is, thus, required to translate only along the vertical direction. The complete movement involves two main parts:

- An ascent phase (A phase—movement along the positive direction of *z*-axis of world frame) consisting of three consecutive steps of amplitude 0.3 m, starting from the initial height of 0.7 m from the ground;
- A following descent phase (D phase—movement along the negative direction of *z*-axis of world frame) consisting of three consecutive steps of amplitude 0.3 m, starting from 1.6 m.

In correspondence to both A and D phase, after any step execution, the vehicle is required to realize the static hovering condition for 5 s.

We took into account path T2 with the intent of analyzing the localization performance by increasing the distance from the AprilTag map. For this reason, in the following, the attention is focused on the positioning error during the hovering phases. In particular, we distinguish between the hovering phase following the take-off maneuver (S0), the hovering phases following each ascent step (A1–A3) and the hovering phases following each descent step (D1–D3). Note that the height imposed on the platform during S0/D3, A1/D2 and A2/D1 is, respectively, the same; while, in correspondence to A3, the UAV is supposed to reach the maximum height of 1.6 m.

### 4.3. Experimental Results

Hereafter, the attention is focused on the error between the position estimation provided by VIO method and the localization output of the VICON motion capture system, meant as ground truth. We discuss the results of the two sets of experiments described in the previous section. In details, in correspondence to both T1 and T2, we investigate the behavior of both QR01 and HR01 during the different flight phases.

#### 4.3.1. T1: Planar Square Trajectory

**Figure 8:**
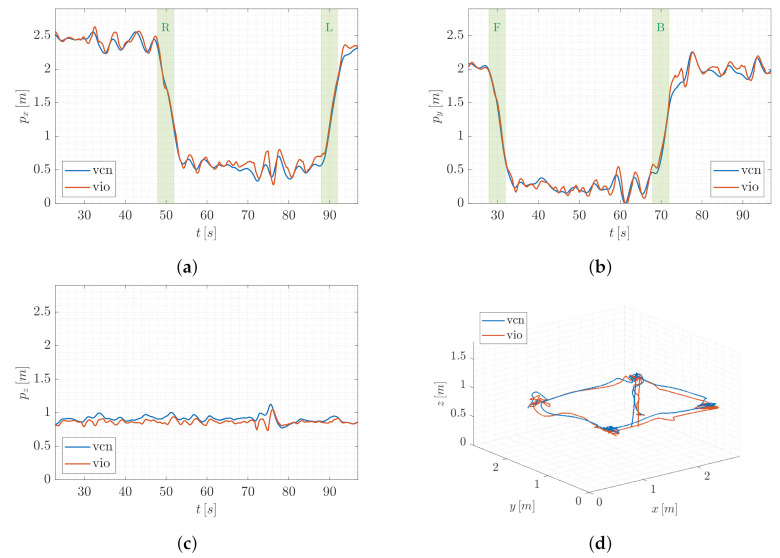
T1-QR01: trend of the UAV position components (**a**–**c**) and 3D path (**d**), comparing the output of VIO localization (vio) and VICON motion capture system (vcn).

**Figure 9:**
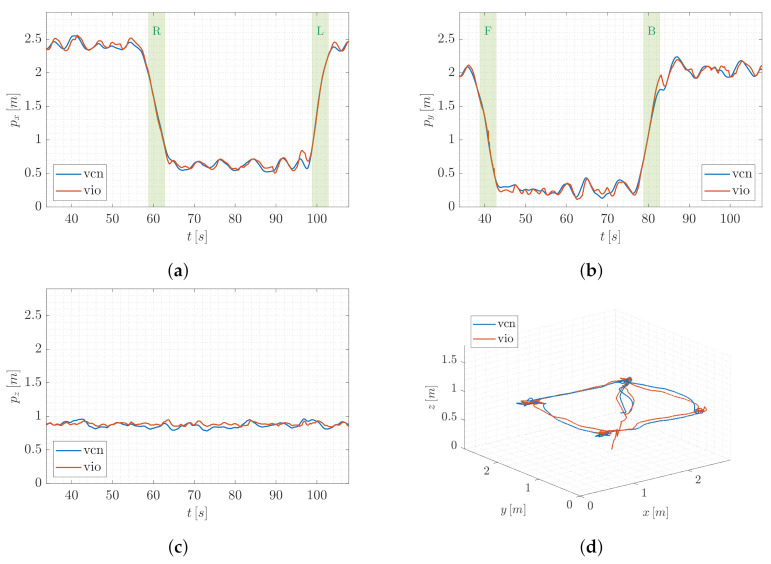
T1-HR01: trend of the UAV position components (**a**–**c**) and 3D path (**d**), comparing the output of VIO localization (vio) and VICON motion capture system (vcn).

In order to first provide a qualitative intuition, without loss of generality, we account for a single trial of T1. Figure 8 and Figure 9 report the trend of the UAV position components in world frame and the 3D path of QR01 and HR01, respectively, comparing the output of VIO localization (vio) and VICON motion capture system (vcn). In Figure 8a,b and Figure 9a,b the principal motion phases (R and L, F and B, respectively) are highlighted (green colored areas). Focusing on these figures, we observe that the outputs of the two compared localization systems are almost overlapped both for QR01 and HR01, validating the goodness of the proposed VIO method in correspondence to rapid longitudinal and latitudinal movements.

For the purpose of also discussing the performance from a quantitative point of view, we introduce the error $e={\left[e_{x}\;\;e_{y}\;\;e_{z}\right]}^{⊤}\in R^{3}$ defined as the difference between the output $p^{\mathrm{vio}}\in R^{3}$ of the VIO localization and the output $p^{\mathrm{vcn}}\in R^{3}$ of the VICON motion capture system. Table 1 reports the mean and standard deviation of such an error computed by taking into account all the trials. We note that the error mean is always included in the interval [−0.11,0.08]m for the quadrotor platform, and smaller values appear in correspondence to the hexarotor platform for which the interval reduces to [−0.05,0.05]m. This is probably due to bigger dimensions (and mass) of the hexarotor UAV which entail higher inertia and thus less vibrations. The performance of HR01 results to also be better in terms of standard deviation, which is not higher than 0.08 m, while the QR01 increases to 0.11 m. Then, we observe that for the QR01 the error component $e_{x}$ is higher during the R and L phases, namely when the movement is performed along the *x*-axis. The same fact occurs for the HR01 in correspondence to the movement along the *y*-axis. Such a fact can be justified by different distributions of the payload onboard the platforms. Indeed, observing, for instance, Figure 5a, it can be noted that the quadrotor payload is more concentrated along the longitudinal direction. Such a direction is orthogonal to the movement direction during R and L phases, thus the inertia turns on to be smaller causing higher vibrations in the UAV dynamics. A similar reasoning can be carried out for the HR01.

**Figure 10:**
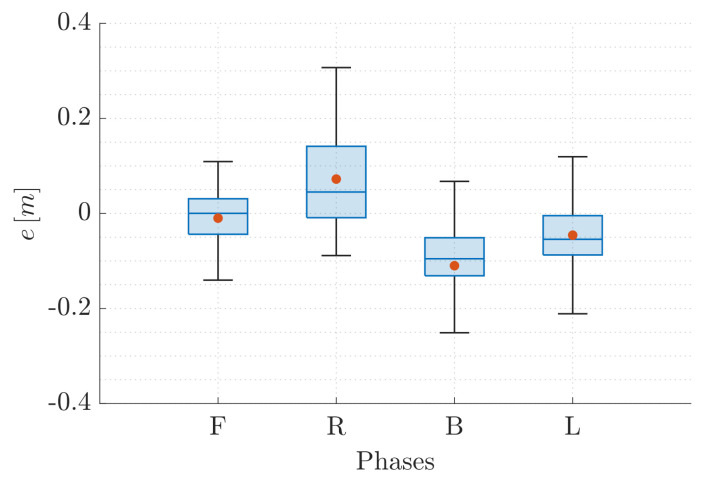
T1-QR01: statistical description of error *e*.

**Figure 11:**
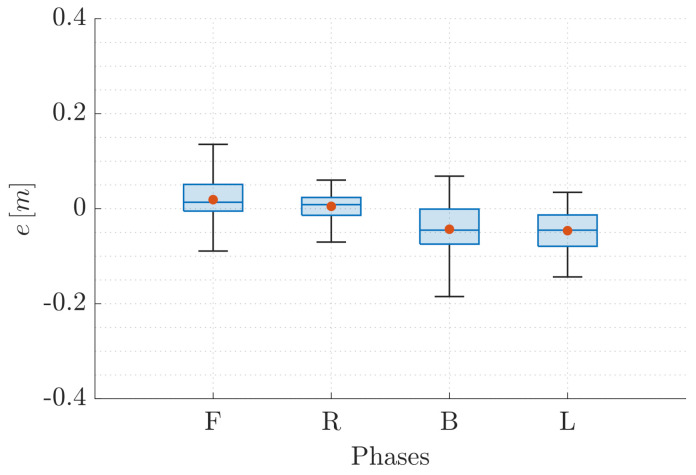
T1-HR01: statistical description of error *e*.

To provide a better insight on the performance of VIO localization method, Figure 10 and Figure 11 report the statistical description of e∈R defined as

(2) $$e=\left\{\begin{array}{cc} e_{x} & \mathrm{for}\;R\;\mathrm{and}\;L\;\mathrm{phases}\\ e_{y} & \mathrm{for}\;F\;\mathrm{and}\;B\;\mathrm{phases} \end{array}\right.$$

accounting for the cumulative results of all the trials wherein T1 is executed by QR01 (Figure 10) and HR01 (Figure 11). For the hexarotor platform, we observe that for all the phases the error does not exceed 0.2 m in terms of absolute value (as confirmed by the amplitude of the total range), and it is generally less than 0.1 m (as confirmed by the amplitude of the interquartile range). We remark that this last value corresponds to the 5% of the total traveled distance during each phase since it is equal to 1.8 m. We also note that the error mean (red point) and median (blue line) approximately correspond and their value is very low in correspondence to the R phase: this can be justified by the payload distribution which can determine a preferential movement in terms of vibration. On the other side, for the quadrotor platform, the boxplots reveal both higher total range and interquartile range which are under 0.3 m and 0.18 m, respectively. The highest error occurs in correspondence to the R phase: this behavior is opposite to the one observed before and it highlights the mechanical differences between the two platforms. We also observe that the gap between the error mean and median is higher, confirming the presence of higher peaks in the signal trend, coherent with the vibrating QR01 dynamics.

To conclude, we focus on the whole 3D path and we emphasize that the discrepancy between VIO and VICON localization is limited to roughly ±0.1 m along all the three axes of the world frame. The T1 results thus highlight the advantages deriving from the density of the designed AprilTag map: the high number of tags allows the UAVs to strongly limit the losses of a position reference, encouraging the employment of the proposed localization method in a real-world application scenario.

#### 4.3.2. T2: Vertical Steps Trajectory

**Figure 12:**
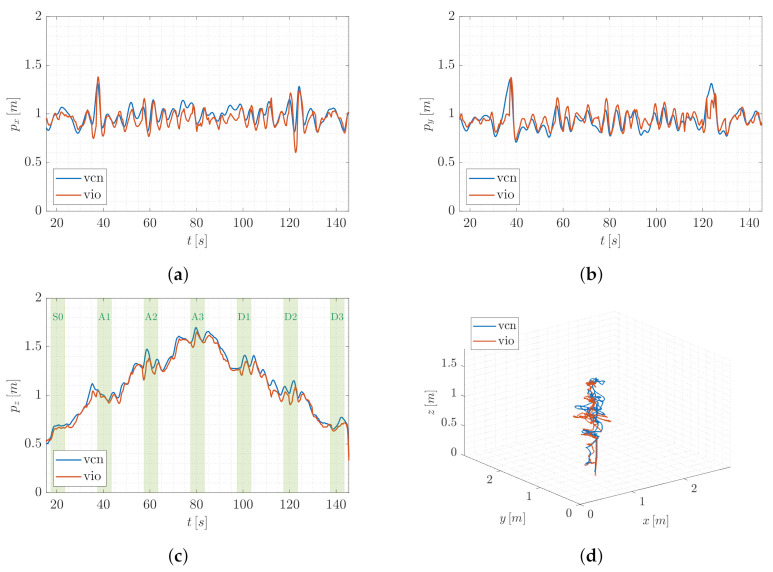
T2-QR01: trend of the UAV position components (**a**–**c**) and 3D path (**d**), comparing the output of VIO localization (vio) and VICON motion capture system (vcn).

**Figure 13:**
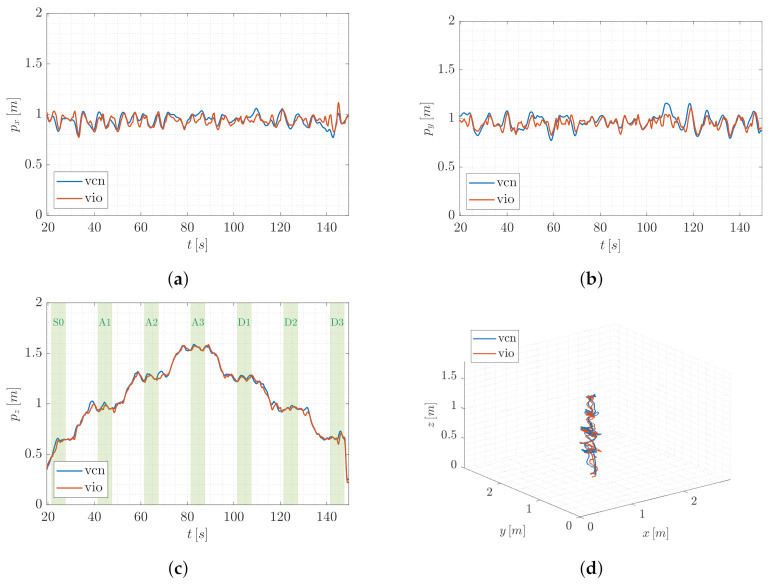
T2-HR01: trend of the UAV position components (**a**–**c**) and 3D path (**d**), comparing the output of VIO localization (vio) and VICON motion capture system (vcn).

As for T1, Figure 12 and Figure 13 depict the trend of the position components in $F_{W}$ and the path followed in the 3D space by the QR01 and HR01, respectively, accounting for a single trial of the T2 set of experiments. Again, we aim at analyzing the performance of VIO localization method (vio) as compared to VICON motion capture system (vcn). In detail, in Figure 12 and Figure 13c, we highlight the different hovering phases taken into consideration (green colored areas, identified sequentially as S0, A1-3 and D1-3), reporting the trend of the position component related to the vertical axis of the world frame (i.e., $p_{z}$). In this case, indeed, the main focus of the analysis is the obtained positioning error in the function of the distance from the ground, namely from the tags.

First, focusing on Figure 12d and Figure 13d, one can observe that for both the aerial platforms all the hovering phases envisaged in the considered trajectory are characterized by some drifts on the (x,y)-plane of the world frame. This is confirmed by the trend of the position components $p_{x}$ and $p_{y}$ in Figure 12a,b (for QR01) and Figure 13a,b (for HR01). In particular, such oscillations with respect to the imposed way-points are wider for the quadrotor platform that drifts till 0.4 m from the desired position, both along the *x*- and *y*-axis of the world frame. This fact is still attributable to its light mass (and inertia) making this platform more prone to vibrations during flight. However, note that the outputs of VIO and VICON system are very closed besides the presence of this oscillatory behavior of the UAVs.

Accounting for a more quantitative analysis perspective, Table 2 reports the mean and standard deviation of the error $e\in R^{3}$ (introduced in the previous section) for the cumulative trials in each hovering phase. We observe that for QR01, the error mean is always included in the range [−0.05,0.07] m. For HR01, instead, the range is [−0.06,0.03] m. These results are almost consistent with the T1 case, confirming the observations in Section 4.3.1 and highlight the better performance of the proposed VIO approach with respect to the strategy outlined in [48] where the reported error range is [−0.6,0.7] m. Furthermore, focusing on the error along the *z*-axis of the world frame, i.e., on $e_{z}$, for both the UAVs we note that the error mean and its standard deviation remain almost inside limited boundaries, meaning that the performance of the proposed localization method do not downgrade in relation to distance from the map, contrarily to the results given in [47,48]. In detail, for QR01 the mean of $e_{z}$ is in the range [0.027,0.042] m and the maximum standard deviation is 0.042 m, while for the HR01 the range of error mean results [−0.008,0.013] m and the maximum standard deviation turns out to be 0.03 m.

**Figure 14:**
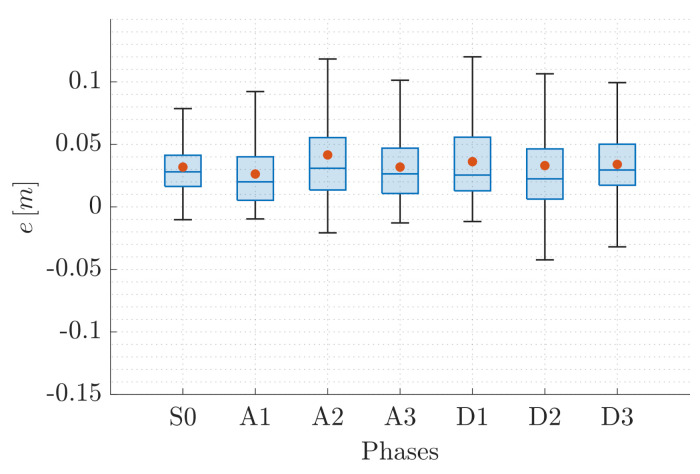
T2-QR01: statistical description of error *e*.

**Figure 15:**
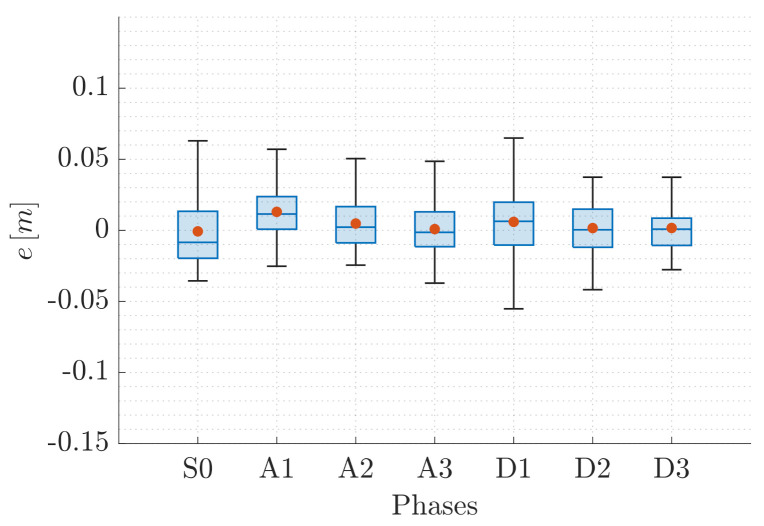
T2-HR01: statistical description of error *e*.

This valuable aspect also emerges from the statistical description of the error $e=e_{z}\in R$, reported in Figure 14 and Figure 15 for QR01 and HR01, respectively. The boxplots show that the maximum value of the error is smaller than 0.12 m for QR01 and 0.08 m for HR01; moreover, the interquartile range is smaller than 0.05 m for both the platforms. We remark that the maximum height reached is approximately 1.6 m, meaning that the error is generally smaller than 5% of the total distance from the ground.

**Figure 16:**
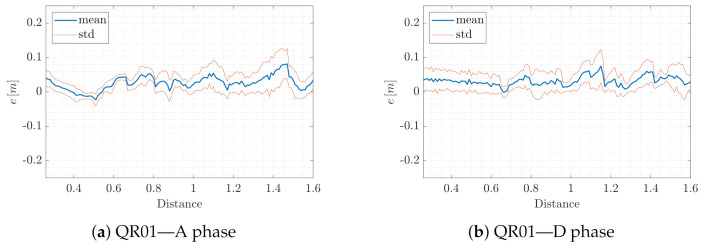

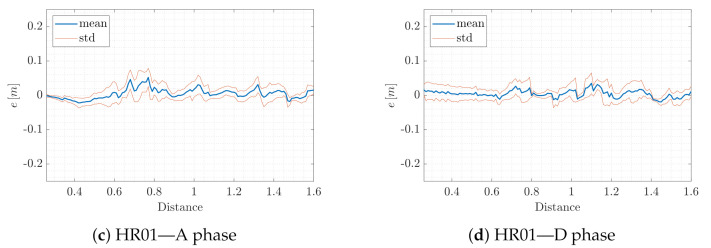
T2-QR01& HR01: mean and standard deviation of error *e* with respect to the AprilTag map distance.

To conclude, we emphasize the independence of the quality of the proposed VIO position estimation from the UAV altitude by evaluating the mean and standard deviation of the error *e* with respect to the AprilTag map distance. In detail, we distinguish between the A and D phase for both QR01 (Figure 16a,b) and HR01 (Figure 16c,d). For both platforms, we observe that the mean of the error *e* is smaller than 0.09 m, during the A and D phases. One can also note that the drops in value of standard deviation are associated with the hovering phases, where more estimates are naturally available for the statistical computation.

The T2 results highlight the advantages deriving from the size heterogeneity of the designed AprilTag map: the different dimensions of the tags allows the UAVs to stably fly at different altitudes in the range [0,1.6]m which represents significant value for indoor industrial applications.

## 5. Conclusions

Motivated by the recent advances in aerial robotics, this work focuses on multi-rotor UAVs involved in indoor applications, proposing an effective localization method based on the VIO approach. The main contribution consists of the definition of an AprilTag map characterized by a high number of narrowly placed tags belonging to four different classes in terms of size. The goal, indeed, is to design a positioning strategy that turns out to be effective for a large number of aerial platform capable of operating at different height from the ground.

From this perspective, the performance of the introduced VIO localization method has been assessed by accounting for a small size quadrotor (QR01 in Figure 5a) and a medium size hexatoror (HR01 in Figure 5b). The results of the conducted experimental tests demonstrate the validity of the outlined solution as compared to the localization output of a motion capture system: the resulting position error is included in [−0.06,0.11]m accounting for translational movement in any direction of the world frame. In addition, the error turns out to be approximately constant by increasing the distance from the tags.

In the future, we plan to improve the AprilTag-based position reconstruction by merging the information deriving from multiple tags suitably weighted according to the view quality of any marker. Moreover, we intend to investigate the VIO localization performance in the presence of occlusions.

## Figures and Tables

**Table 1:** T1-QR01 & HR01: error mean and standard deviation of the components of the error e (in cm).

		Phase			
		F	R	B	L
QR01	$e_{x}$	2.313±4.351	7.228±10.728	−3.115±6.399	−4.572±9.183
	$e_{y}$	−0.994±5.458	0.802±5.155	−10.996±9.928	−3.948±7.108
	$e_{z}$	4.193±2.487	6.115±2.673	7.369±7.813	4.940±6.453
HR01	$e_{x}$	3.178±4.654	0.498±2.707	−1.653±4.701	−4.622±4.192
	$e_{y}$	1.902±4.718	0.172±4.854	−4.321±4.997	−1.332±6.114
	$e_{z}$	4.443±2.864	0.148±6.617	1.877±7.589	2.532±6.482

**Table 2:** T2-QR01 & HR01: error mean and standard deviation of the components of the error e (in cm).

		Phase						
		S0	A1	A2	A3	D1	D2	D3
QR01	$e_{x}$	−0.836±3.402	2.621±5.704	1.787±6.474	6.328±7.917	2.025±5.546	0.381±8.554	−2.304±3.949
	$e_{y}$	−2.285±4.251	−1.245±6.080	−2.832±6.525	−4.645±6.835	−1.348±5.094	−0.127±7.979	−0.005±4.045
	$e_{z}$	3.186±2.076	2.631±2.697	4.157±4.173	3.187±2.859	3.622±3.143	3.305±4.000	3.402±2.413
HR01	$e_{x}$	−5.655±2.789	−0.704±3.811	1.930±1.974	2.975±2.824	1.700±4.338	−0.158±3.876	−4.079±4.122
	$e_{y}$	1.968±3.378	−1.516±4.789	−1.985±3.380	−1.508±2.757	0.539±4.692	0.543±4.474	1.024±3.655
	$e_{z}$	−0.075±2.399	1.299±1.815	0.477±1.664	0.084±1.635	0.592±2.910	0.157±1.763	0.161±1.736

## Data Availability

Not applicable.
